# Does remote ischaemic conditioning reduce inflammation? A focus on innate immunity and cytokine response

**DOI:** 10.1007/s00395-021-00852-0

**Published:** 2021-02-24

**Authors:** Lucie Pearce, Sean M. Davidson, Derek M. Yellon

**Affiliations:** The Hatter Cardiovascular Institute, 67 Chenies Mews, London, WC1E 6HX UK

**Keywords:** Cytokines, Inflammation, Myocardial infarction, Remote ischaemic conditioning

## Abstract

The benefits of remote ischaemic conditioning (RIC) have been difficult to translate to humans, when considering traditional outcome measures, such as mortality and heart failure. This paper reviews the recent literature of the anti-inflammatory effects of RIC, with a particular focus on the innate immune response and cytokine inhibition. Given the current COVID-19 pandemic, the inflammatory hypothesis of cardiac protection is an attractive target on which to re-purpose such novel therapies. A PubMed/MEDLINE™ search was performed on July 13th 2020, for the key terms RIC, cytokines, the innate immune system and inflammation. Data suggest that RIC attenuates inflammation in animals by immune conditioning, cytokine inhibition, cell survival and the release of anti-inflammatory exosomes. It is proposed that RIC inhibits cytokine release via a reduction in nuclear factor kappa beta (NF-κB)-mediated NLRP3 inflammasome production. In vivo, RIC attenuates pro-inflammatory cytokine release in myocardial/cerebral infarction and LPS models of endotoxaemia. In the latter group, cytokine inhibition is associated with a profound survival benefit. Further clinical trials should establish whether the benefits of RIC in inflammation can be observed in humans. Moreover, we must consider whether uncomplicated MI and elective surgery are the most suitable clinical conditions in which to test this hypothesis.

## Background: challenges and new directions in RIC

Bringing the promise of remote ischaemic conditioning (RIC) to fruition in the clinical arena, remains a major challenge [23, 58]. RIC involves the sequential occlusion and reperfusion, of an arterial vessel distant to the target organ. It has demonstrated multi-organ benefit and cross-species cardiovascular protection in studies of ischaemia [13], and is highly effective in preventing damage in animal models of myocardial infarction [63]. However, large-scale trials in humans with ST-elevation myocardial infarction (STEMI) have proved inconclusive, with respect to traditional outcome measures of myocardial infarct size, heart failure and survival [36, 48, 58]. RIC confers cardioprotection via a combination of humoral and neuronal pathways. These link the protective, “conditioning” response to ischaemia induced in the remote vascular bed, to the target tissue at risk of severe ischaemia and reperfusion (I/R) injury [7, 80]. Whilst many potential humoral factors have been proposed such as nitric oxide (NO) and nitrite, adenosine, stromal-derived factor 1α (SDF-1α) and glucagon-like peptide-1 (GLP-1); the underlying immunological pathways remain poorly defined [8, 27, 58, 80, 118].

The effectiveness of RIC in preventing myocardial I/R injury in humans has been assessed in numerous studies, most notably the CONDI-2/ERIC-PPCI trial, an international, prospective, single-blind, randomised controlled outcome trial in 5,401 patients with ST-elevation myocardial infarction (STEMI) undergoing primary percutaneous coronary intervention (PPCI), in which no improvement in clinical outcomes (cardiac death or hospitalisation for heart failure) were seen after 12 months [48]. Importantly, however, no harmful effects were seen. Many theories have sought to explain why the success of RIC in animal models has not been directly translatable to humans [58, 64]. One important observation, highlighted in two recent articles, is that the population studied in the CONDI-2/ERIC-PPCI trial may not have been significantly ‘high-risk’ enough, to demonstrate an improvement in the primary outcome measures of infarct size and survival [11, 49, 65]. It is likely, however, that a plethora of factors make the human model of cardioprotection more complex than the animal population, who are often devoid of chronic endothelial dysfunction and medical co-morbidities [57, 65]. Moreover, the timing and size of an experimentally induced infarct in animals can be carefully predicted and is reproducible. The key to translation may lie in better understanding of the underlying mechanisms, and how these can be applied to human physiology in individual conditions.

**Fig.1:**
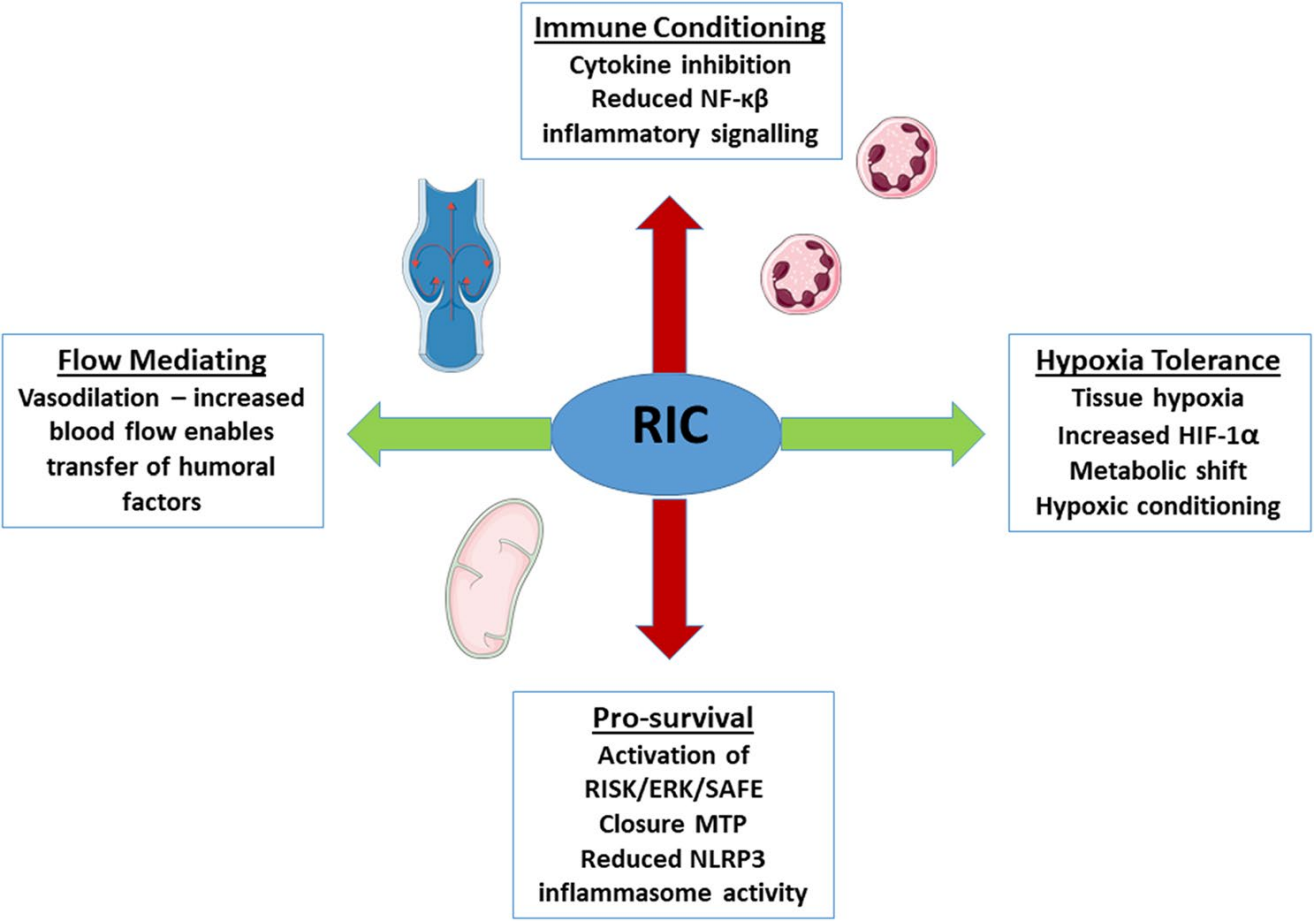
RIC mediates inflammation in vivo by immune conditioning and cytokine inhibition, anti-apoptotic pathways and the reduction of NLRP3 inflammasomes, (pyroptosis). Decreased oxygen tension adjusts cell metabolism and limits apoptosis. Shear stress induces flow-mediated dilatation, which enables the transfer of exosomes carrying anti-inflammatory, chemo-active compounds. *RISK* reperfusion injury salvage kinase, *ERK* extracellular signal related kinase, *SAFE* survivor activating factor enhancement, *MTP* mitochondrial transition pore, *HIF-1α *hypoxia inducible factor 1 alpha, *NFκβ *nuclear factor kappa beta

We have embarked upon a new era of inflammation in cardioprotection. With the arrival of novel diseases such as COVID-19, and its associated cardiovascular complications, there is a need to re-fashion current cardioprotective strategies. Moreover, the pandemic has identified the need to further investigate the effects of infection on the heart in addition to ischaemia. Whilst many reviews have considered RIC as an infarct limiting intervention, the effects on the innate immune system are less well documented. We present this review of the anti-inflammatory effects of RIC, and the implications for future organ-protective therapies (Fig. 1).

## The inflammatory hypothesis of organ protection

**Fig. 2:**
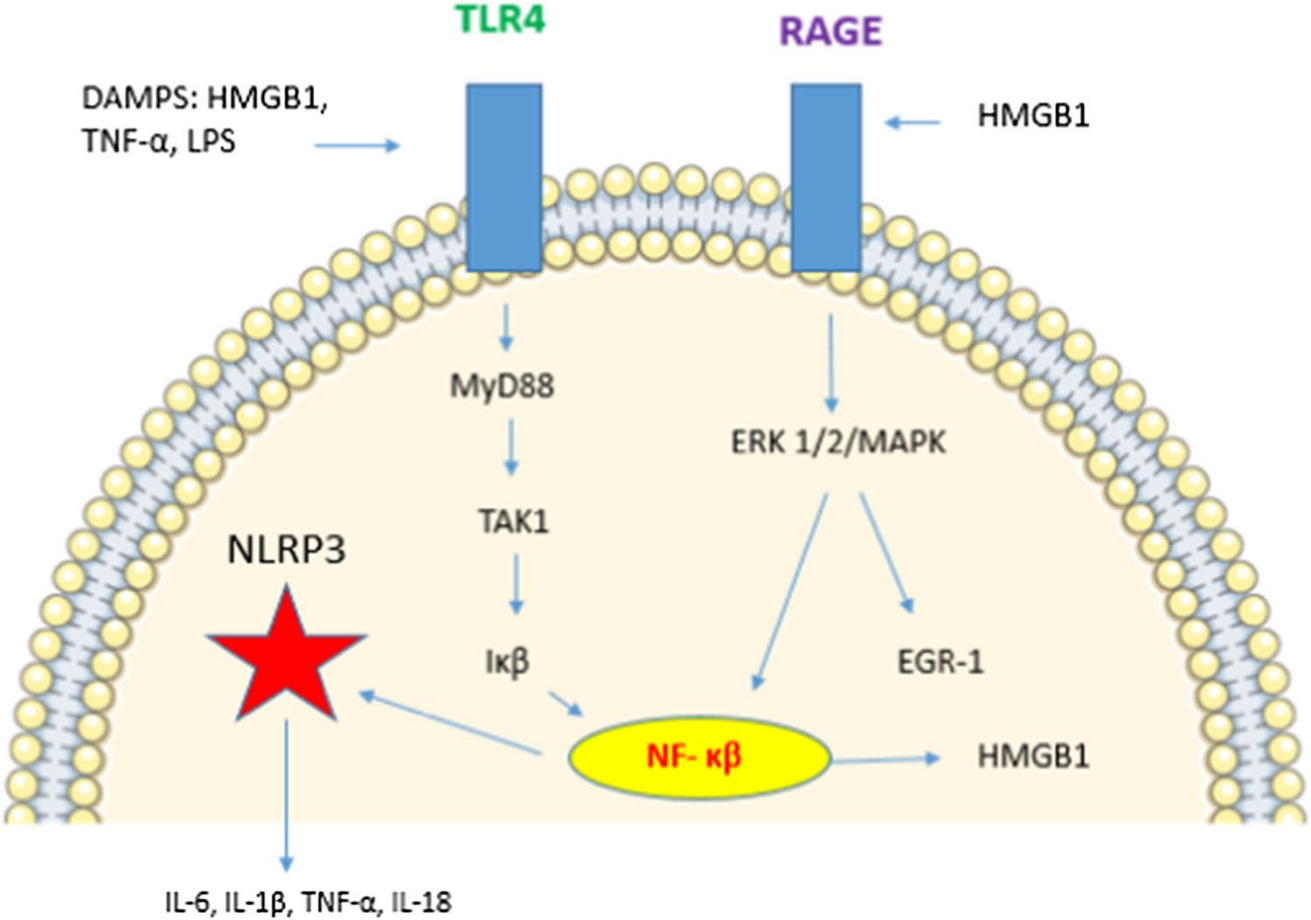
Cytokine release in inflammation is mediated by DAMPS binding to TLR4/RAGE receptors on the cell membrane. Both pathways activate NF-κB and NLRP3 inflammasome production, resulting in secretion of pro-inflammatory cytokines [75, 150]. RAGE results in further production of HMGB1 [151]. NLRP3 inflammasome activation results in caspase mediated cell death [119]. Several studies haves demonstrated that RIC modulates NF-κB activity via in both ischaemia and endotoxaemia [74, 76, 92, 117, 130]. *TLR4* Toll-like receptor 4, *DAMPS* damage associated molecular patterns, *HMGB1 *high mobility group box 1, *TNF-α *tumour necrosis factor alpha, *LPS* lipopolysaccharide, *RAGE* Receptor for advanced glycation end-products, *ERK* extracellular signal related kinase, *MAPK* mitogen activated protein kinase, *EGR-1* early growth response 1, *TAK1* transforming factor-β-activated kinase 1, *Iκβ *inhibitor kappa beta kinase, *NF-κβ *nuclear factor kappa beta

The ‘Inflammatory Hypothesis’ is a term used to define the role of the innate immune system in I/R injury. Following reperfusion in myocardial infarction, acute inflammation contributes to endothelial dysfunction, the development of cardiac failure and poor left ventricular remodelling [99, 109, 157]. Such damage persists well after the initial ischaemic insult has ended, and the infarct related territory has been reperfused [148]. Upon reperfusion, resident immune cells detect the presence of danger-associated molecular proteins (DAMPs) and necrotic tissue in the area of infarction [157]. DAMPS combine with other ‘*alarmin*’ molecules, such as high mobility group box one protein (HMGB1), extracellular DNA and histones, to trigger the secretion of pro-inflammatory cytokines via the cell-mediated nuclear factor kappa beta (NF-κB) pathway (Fig. 2) [128, 157]. Macrophages, which engage with DAMPs via toll-like receptors (e.g. TLR4), are also responsible for the synthesis of pro-inflammatory molecules including cytokines and the NLRP3 inflammasome (Fig. 2).

Following myocardial ischaemia, four varieties of programmed cell death are observed, including apoptosis, necrosis, necroptosis and pyroptosis [21, 61]. Apoptosis is triggered by death receptors (DR) and intracellular signals, and does not induce the release of cellular contents beyond the confines of the cell. It is largely mediated via caspase-8, 9 and Bc12. Both necroptosis and pyroptosis, enhance inflammation by facilitating the release of interleukins such as IL-1β and IL-18. [61, 135]. Pyroptosis is a type of programmed cell death (PMD) which is closely related to activity of the NLRP3 inflammasome and NF-κβ. Here, activated caspase-1 facilitates the release of interleukins via the Gasdermin (GSDMD) membrane pore. Not only does inflammasome activation contribute to cytokine release and extent of inflammation, but also to infarct size, following myocardial ischaemia [29, 135]. The latter is, therefore, an attractive target for cardioprotection [1]. Whilst there are clear associations between RIC and anti-apoptotic pathways [122], it remains unclear whether remote conditioning can directly limit pyroptosis (and at which step). There is, however, evidence for bi-directional cross-talk between caspase-1 and caspase-8 (anti-apoptotic) suggesting that inhibition of apoptosis might also influence other forms of cell death [29].

The microvasculature has a central role in mediating inflammation during I/R, and endothelial cells are notoriously more resistant to hypoxia than other cell types [109, 137]. They are, however, sensitive to the presence of reactive oxygen species (ROS) and the changes in NO metabolism that accompany an ischaemic challenge [4, 47, 125]. The coronary endothelial system has an important role in detecting mechanical and flow-mediated changes post-infarct, in addition to mediating the vascular immune response. In the case of myocardial infarction, within the coronary arteries, exposed atherothrombotic plaque causes cells of the innate immune system to migrate to the inflamed vessel, prompting further cytokine release. Increased neutrophil recruitment to the area of vascular inflammation is mediated by cytokines, the complement cascade (including IL-8; C5) and directly via ROS [109, 125]. Polymorphonuclear neutrophils (PMNs) are attracted to the endothelium via selectins and proceed to adhere and transmigrate into the microvasculature by binding to integrins and ICAM adhesion molecules [115].

As the inflammatory hypothesis has evolved, immune-modulating therapies have been extensively investigated in both myocardial and cerebral infarction, and have finally met with some recent success. The CANTOS trial demonstrated that the IL-*1*β blocker, Canakinumab, was able to reduce the risk of future coronary and cerebral atherosclerotic events [120]. This benefit was, however, associated with a mild increase in fatal infection, which should not be disregarded. Nevertheless, the CANTOS trial was important in measuring outcomes of inflammation in cardiac protection, and included measurements of cytokines involved in IL-6 signalling and C-reactive protein (CRP) [120]. Both biomarkers were deemed to be prognostic, with respect to the primary end-point of non-fatal atherosclerotic events. This might suggest that cytokines are valuable biomarkers in predicting future adverse events in patients with myocardial infarction [120]. As further justification to pursue cytokine inhibition in I/R, two recent clinical studies of STEMI patients have demonstrated that IL-6 and IL-8 are associated with worsening clinical outcome [42, 129].

In the VCUART3 trial, the recombinant interleukin-1 receptor antagonist, Anakinra reduced CRP levels at 14-days post STEMI, and significantly reduced mortality and re-hospitalisation. Unlike CANTOS, there was no increase in severe infection reported following administration [2]. Similarly, IL-1 inhibition was well tolerated when administered subcutaneously to patients with acute cerebral infarction, in the SCIL-STROKE trial. In this study, Anakinra significantly lowered levels of IL-6 and plasma CRP (p < 0.001) [132], suggesting that it is a key mediator of the inflammatory response in cerebral ischaemia [72]. Although experimental results are promising in both myocardial and cerebral infarction, immune modulation is not currently used as routine in STEMI patients. Inhibition of individual cytokines must be carefully risk stratified, due to the close association between innate immune suppression and the development of humoral immunity [126].

## Cytokines in myocardial infarction and infection

The pro-inflammatory cytokines released in response to myocardial infarction include IL-1α, IL-1β, IL-6, TNFα, IL-8, IL-18 and small chemokine molecules such as monocyte chemoattractant protein 1 (MCP-1). These cytokines are released by a mixture of damaged cardiomyocytes, macrophages and activated endothelium [109, 115]. The primary aim of cytokine release is to activate and attract immune cells to the area of inflammation, to enable the removal of damaged products via phagocytosis [5]. In the infarcted heart, cytokine release is triggered by TLR4 signalling pathways, the activation of NF-κB in circulating macrophages and by reactive oxygen species (ROS), which interact with IL-6. The resultant release of IL-1β further stimulates additional pro-inflammatory molecules [17, 107]. Cytokine ‘cross-talk’ exists between immune cells and the activated endothelium, which maintains the amplitude of the acute inflammatory response [133].

There are two distinct phases of inflammation following myocardial infarction: an initial, pro-inflammatory phase in which damaged cells and debris are eliminated, and a second, anti-inflammatory reparative phase leading to wound healing and scar formation. Cytokines also have a key role in tissue repair. IL-6, (in a second window of cytokine release), IL-10, transforming growth factor beta (TGF-β) and a sub-population of T-lymphocytes known as ‘T Regulatory cells’ (Treg) have all been associated with supressing the pro-inflammatory response and steering the immune system towards repair and resolution following I/R [99, 107]. Macrophages expressing altered interleukin signals (such as IRAK-M) are able to downregulate other macrophages, contributing towards anti-inflammatory ‘stop’ signals [107, 155]. For suitable healing to take place, the amplitude of the initial macrophage activation syndrome must not outweigh that of regulatory immune cells. In myocardial infarction, cytokine release predominantly occurs on the border of the infarct zone, but can also be present in non-ischaemic tissue [17].

The cardiovascular system is vulnerable to inflammatory insult induced by infection and cytokine damage, including viral myocarditis, septic cardiomyopathy and recently the acute cardiovascular syndrome of COVID-19 [40, 68, 86]. The latter encompasses thrombotic acute coronary syndromes, myocarditis and pulmonary emboli, amongst other complications of pre-existing cardiac disease [40]. The cytokine response to infection is similar in the acute phase to myocardial infarction, and is triggered by DAMPS and danger signals following pathogenic invasion (stimulating IL-6, TNFα and IL-1β). In some individuals, following exposure to endotoxins or viral antigens, the innate immune system becomes hyper-active and a ‘*cytokine storm’* develops. Here, further cytokines are secreted (IL-17, IL-8, G-CSF, MCP-1, CCL1-3, IFN-*y*) and re-circulated via the dysfunctional endothelium. The following present an in-depth review of cytokine response during infection [84, 112, 153]. This has been a topic of much importance in COVID-19 and culminates in pan-vascular and multi-organ damage [102].

Given that multiple pathologies affect the heart in this pandemic era, it is important to consider protective strategies which will target the inflammation of both I/R and pathogenic invasion. Below we will consider the evidence for RIC as a cytokine mediator in both animal and human studies.

### Animal studies of RIC and inflammation

Table.1 demonstrates the animal studies of RIC and cytokine release, performed within the last 5 years, following myocardial infarction and reperfusion [9, 15, 33, 114, 136, 141, 152] Across several studies, RIC was associated with reduced levels of the pro-inflammatory cytokines, IL-1β, TNF-α and HMGB1 following reperfusion [114, 152]. Likewise, RIC applied 24h prior to myocardial I/R, appeared to increase levels of the protective cytokine IL-10 [15], which governs the amplitude of the cytokine response [34, 127]. In vivo, this increase in IL-10 was STAT5 mediated [15]. STAT5 is linked to the survivor activating factor enhancement pathway (SAFE) and operates downstream of JAK (Janus Kinase) in human myocardial injury [66]. Similarly, previous literature has discussed the protective effects of IL-10, limiting I/R injury via STAT3 [44, 79, 100]. In another study, RIC was associated with an increase in IL-6 (which the authors propose has reparative function within the infarcted myocardium) via early growth response protein 1 (EGR-1), a molecule upstream of many apoptotic pathways [9]. In animal models of myocardial infarction, RIC combined with other therapies such as sevoflurane post-conditioning (anaesthesia following ischaemia and prior to onset of reperfusion) vagal nerve stimulation or atorvastatin (HMG-CoA reductase inhibitor), provided additive organ protection and reduced inflammation [33, 141, 152].

**Table 1:** Animal models of RIC and cytokine release in myocardial infarction (2015–20)

Authors	Model of I/R	RIC methods (H/R)	Cytokine response	Other findings
Billah et al. [9]	Myocardial infarction (LAD) male SD rats	5/5 min (three cycles) hind limb vs control (I/R)	Inhibition of EGR-1 in vivo significantly attenuated RIC-mediated increase in IL-6 (*p* < 0.05) Inhibition of EGR-1 did not significantly attenuate RIC-mediated changes to IL-1β (p > 0.05)	Inhibition of EGR-1 in vivo significantly attenuated RIC-induced reduction in infarct size (p < 0.001)
Tolkmitt et al. [136]	Global myocardial I/R (90/90), male Lewis rats	5/5 min (three cycles) hind limb vs isoflurane + buprenorphine anaesthesia vs control (I/R) vs sham	Neither RIC nor anaesthesia significantly reduced perfusate levels of the cytokine TNF-α	RIC and onset of reperfusion compared with controls (p < 0.001; p < 0.05) RIC and anaesthesia independently increased coronary flow (%) prior to onset of reperfusion compared with controls (p < 0.001)
Pilz et al. [114]	Myocardial infarction (LAD) 30 min + 14 days reperfusion, male SD rats	5/5 min (three cycles) hind limb vs controls (I/R) vs sham	RIC significantly reduced expression of TNF-α vs controls ex vivo (*p* < 0.05) RIC significantly reduced expression of IL-1β vs controls (p < 0.01) RIC significantly reduced expression of TLR4 vs controls (p < 0.05)	RIC significantly improved LVEF (%) and reduced scar formation (*p* < 0.01) in vivo by TTE RIC significantly improved CO ex vivo vs controls (p < 0.05)
Chen et al. [15]	Myocardial infarction (LAD) (30/180) in mice and Stat5 knock-out mice	5/5 min (three cycles) surgical femoral artery occlusion vs control (I/R) vs sham	IL-10 levels were significantly increased in Stat5 + VE mice undergoing RIC + I/R vs controls (I/R) (p < 0.05)	RIC significantly reduced infarct size (%) vs controls (p < 0.01) in Stat5 + VE mice RIC did not significantly reduce infarct size (%) vs controls in Stat5 knock-out mice (p > 0.05) RIC significantly reduced apoptosis (% TUNEL + ve cells) vs controls in Stat5 + ve mice (p < 0.01). These effects were lost in Stat5 KO animals RIC significantly reduced caspase levels vs controls in Stat5 + ve mice (p < 0.05) HIF-*1a* levels were significantly increased in Stat5 + ve mice undergoing RIC + I/R vs controls (*p* < 0.001)
Zhang et al. [152]	Myocardial infarction (LAD) (30/120 min), male SD rats	5/5 min (four cycles) vs controls (sham), I/R, post-conditioning with sevoflurane (SPC) and SPC + RIPC	Pro-inflammatory cytokines IL-6,8 and TNF-*a* increased after I/R vs controls (*p* < 0.01) RIC significantly reduced serum concentrations of IL-6,8 and TNF-α vs I/R (*p* < 0.05) Cytokine suppression effect was greatest when RIC was combined with SPC vs I/R (*p* < 0.05)	LVEDP was significantly improved in animals undergoing RIC vs I/R alone (p < 0.05) Infarct size was significantly reduced in animals undergoing RIC vs I/R, and SPC + RIC vs I/R (*p* < 0.05) RIC and RIC + SPC significantly reduced expression of TLR4, NF-*kB*, HMGB1 (p < 0.05)
El Desoky et al. [33] (Animal experiment)	Myocardial infarction (LAD occlusion) 30/120 min, male New Zealand white rabbits	3 cycles of single hindlimb IR (5/5 min)	RIC in combination with atorvastatin provided 6-weeks prior to I/R, significantly reduced serum levels of IL-6, TNF-α, and CRP (p < 0.05) vs I/R controls RIC alone significantly reduced serum levels of TNF-α (*p* < 0.05) vs I/R controls	Both RIC alone, and in combination with atorvastatin, significantly increased serum levels of NO (p < 0.05) Atorvastatin + RIC significantly reduced levels of CRP vs I/R controls (p < 0.05)
Wang et al. [141]	Myocardial infarction (LAD) ligation, 30/120 min male SD rats, RIC and VSPer	10 min bilateral hind limb ischaemia for RIC group, VSPer = 30 min vagal stimulation	RIC and RIC + VSPer significantly reduced levels of TNF-α, HMGB1, at up to 120 min of reperfusion vs I/R controls (*p* < 0.05) RIC and RIC + VSPer significantly reduced levels of ICAM-1, IL-1, IL-6 vs I/R controls (*p* < 0.05) RIC and RIC + VSPer significantly increased levels IL-10 vs sham (no I/R) p < 0.05	RIC and RIC + VSPer significantly reduced infarct size (%) vs I/R controls (p < 0.05) RIC and RIC + VSper significantly reduced serum troponin post-infarct (ng/ml) vs IR controls (p < 0.05)

*I/R* ischaemia/reperfusion, *LAD* left anterior descending, *H/R *hypoxia/reoxygenation, *EGR* early growth factor response, (a transcription factor involved in the inflammatory response), *LVEDP* left ventricular end diastolic pressure, *LVEF* left ventricular ejection fraction, *CO* cardiac output, *KO* knock-out, *SD* Sprague Dawley, *SPC* sevoflurane pre-conditioning, *NO* nitric oxide, *VSPer* Per conditioning by vagal stimulation, *UL* upper limb, *LPS* lipopolysaccharide, *dMCAO* distal middle cerebral artery occlusion, *CKD* chronic kidney disease, *CABG* coronary artery bypass graft, *IL* interleukin, *TNF-α* tumour necrosis factor alpha, *cTn* cardiac troponin, *MCP* monocyte chemoattractant protein;* + VE* positive

RIC has proven effective at attenuating pro-inflammatory cytokine release in animal models of cerebral infarction, renal, pulmonary and hepatic reperfusion injury [31, 70, 83, 145, 156]. In a population of aged rats undergoing middle cerebral artery occlusion (mCAO), RIC significantly reduced levels of IL-1, IL-6 and IFN-γ in both plasma and the brain, whilst reducing the expression of hypoxia inducible factor (HIF-1α). Although HIF-1α is linked to cardioprotection by stimulating pro-survival pathways, it can equally induce a shift towards anaerobic glycolysis in macrophages resulting in increased cytokine manufacture [19]. It is previously discussed that the role of HIF-1α in cardioprotection is not fully understood, although deficiency in mice appears to dampen a reduction in infarct size [59]. In a murine model of hepatic I/R injury, RIC significantly reduced levels of intrinsic liver enzymes, IL-6 and TNF-α [150]. Furthermore, this anti-inflammatory effect was mediated by the HMGB1/TLR4/NF-κB pathway, an established mechanism of cytokine release [150]. Pro-inflammatory pathways involving NF-κB, including notch signalling [67, 121] will be considered in greater detail below.

RIC reduces inflammatory cytokine levels and improves survival in rodent models of lipopolysaccharide (LPS) induced endotoxaemia [68, 74, 76]. In mice receiving three cycles of hind limb I/R (10 min ischaemia/10 min reperfusion) prior to LPS exposure, there was a significant survival benefit from RIC (10% of the control group survived vs 60% of the intervention group; *p* < *0.001*). In the same study, histology revealed a reduction in the diffuse parenchymal pulmonary inflammation associated with LPS-induced acute lung injury, and a reduction of cytokines in bronchoalveolar fluid (TNF*-α*, IL-1β and IL-6). It was further demonstrated that RIC mediates cytokine reduction via a downregulation of NF-κB and myeloperoxidase (MPO) pathways [76]. MPO is associated with increased neutrophil influx to areas of inflammation and, therefore, promotes the release of pro-inflammatory cytokines from neutrophils, which are sentinel cells in the inflammatory response [78].

LPS induces a potent inflammatory state, and causes cytokines and alarmins (e.g. HMGB1, HSP70, histones) to be released in response to infection. Bacterial DAMPs/LPS trigger NF-*kB* activity via TLR4, which potentiates further inflammasome and cytokine release (Fig. 2) [77]. In septic cardiomyopathy, the myocardial depressant cytokine HMBG1 is central to the stimulation of inflammation and upregulates the coagulation cascade [68, 94]. There is also evidence to suggest that the cytokines IL-1, TNF-*α*, and IL-6 play a pivotal inflammatory role in endotoxaemia, and this has also been observed in COVID-19 hyper-inflammation [113]. IL-6 in particular, can target the vasculature to induce vasodilatation and disruption of endothelial tight junctions, which results in capillary leak and circulatory collapse [138]. In view of the above, RIC may be a novel treatment modality with cytokine-modulating potential, without the associated side effects of immune-modulating pharmacotherapy.

### Human studies of RIC and cytokine response

Contrary to the findings in animal models, the majority of recent randomised control clinical trials have been unable to demonstrate a clear effect of RIC on pro-inflammatory cytokine release (Table.2) [37, 39, 105, 106, 108, 149, 158]. However, to date, cytokines have predominantly been measured in small studies only (less than 100 participants). Despite this, the largest two trials (*n* = 65, *n* = 90 participants) demonstrated cytokine attenuation in the treatment group undergoing RIC prior to off-pump CABG and colorectal surgery, respectively [53, 140]. In the latter study, levels of IL-1β and TNF-α were significantly reduced for up to 3 days post-operatively (*p* < 0.01) in patients receiving RIC, compared with controls. Surgery was performed for a range of pathologies, including colorectal neoplasm [53]. Considering that RIC has conferred a profound survival benefit in animal models of intra-abdominal injury, it could be suggested that the inflammatory benefits in humans might differ between clinical conditions [110].

**Table 2:** Clinical RCTs of RIC and cytokine release (2015–20)

Authors	Study design	Participants	Method of RIC	Inflammatory findings
Godskesen et al. NCT02445365 [39]	Randomised, single-blinded, sham- controlled trial	Patients with ulcerative colitis and moderate disease activity underwent 10 days of RIC vs sham controls (*n* = 22)	4 alternating cycles of 200 mmHg arm blood pressure cuff inflation and deflation (5/5 min)	No significant differences observed in plasma cytokine levels between groups (*p* > 0.05) No significant differences observed in neutrophil infiltration on rectal biopsy (*p* = 0.85) No significant differences observed in plasma CRP levels (*p* = 0.63)
Oh et al. KCT001384 [108]	Randomised, double-blinded control trial	Patients undergoing RIC prior to shoulder surgery in the beach chair position (cerebral hypo perfusion) vs controls (*n* = 63)	3 alternating cycles of thigh blood pressure cuff inflation (to 2 × baseline BP value) and deflation (5/2 min)	RIC increased cerebral oxygen saturations during surgery (*p* = 0.007) No significant differences observed in levels of IL-1β, IL-6, IL-10 and transforming growth factor
Wang et al. NCT03340181 [140]	Randomised, single-blinded control trial	Patients with IHD undergoing RIC prior to off-pump CABG vs controls (*n* = 65)	4 cycles UL ischaemia with inflation (to 40 mmHg greater than baseline BP) and deflation (5/5 min)	RIC significantly reduced plasma levels of troponin T (*p* < 0.05) RIC significantly reduced levels of IL-6, IL-8 and TNF-α (*p* < 0.05) and increased levels of HIF-1α (*p* < 0.05)
Zwaag et al. NCT02602977 [158]	Randomised, single-centre control trial	Healthy male volunteers undergoing RIC for 6 days + RIC 40 min prior to LPS challenge vs RIC 40 min before LPS alone vs controls receiving LPS (*n* = 30)	4 cycles of UL ischaemia with cuff inflation to 250 mmHg and deflation (5/5 min)	No significant differences observed in levels of TNF-α, IL-6, IL-8 and MCP-1 (*p* > 0.10)
Zapata-Chavira et al. [149]	Randomised, control trial	Patients with CKD undergoing RIC prior to heterotopic renal transplant vs controls (*n* = 29)	Bilateral thigh cuff inflation to 200 mmHg (10 min)	Significantly higher levels of TNF-α and IL-6 in the RIC group (*p* < 0.05)
Ney et al. Sub-analysis of NCT01067703 RIPheart [106]	Randomised, double-blind, multi-centre control trial (sub-analysis)	Patients with IHD undergoing RIC prior to CABG with propofol anaesthesia vs controls (*n* = 40)	4 cycles of UL ischaemia with cuff inflation to 200 mmHg (or > 15 mmHg higher than baseline whichever greater) and deflation (5/5 min)	No significant differences observed in post-operative troponin levels or cytokine levels (*p* > 0.05)
Nederlof et al. NTR2915 [105]	Randomised, double-blind single-centre control trial	Male patients with IHD undergoing RIC prior to CABG with sevoflurane anaesthesia vs controls (*n* = 29)	3 cycles of UL ischaemia with cuff inflation to 200 mmHg and deflation (5 min)	No significant difference in CtnT levels at 24 h (*p* = 0.76) between groups No significant differences observed in levels of cytokines before and after RIC (*p* > 0.05)
Gedik et al. NCT01406678 [37]	Randomised, double-blind control trial (sub-analysis)	Patients with IHD undergoing RIC prior to CABG with sufentanil anaesthesia vs controls (*n* = 46)	3 cycles of UL ischaemia with cuff inflation and deflation (5/5 min)	RIC reduced troponin levels at 72 h post-op (*p* < 0.05) Significantly higher levels of IL-1α in the RIC group (*p* < 0.05), no differences observed in other pro-inflammatory cytokines (*p* > 0.05)
He et al. [53]	Randomised, double-blind, control trial	Patients 65–75 years undergoing RIC prior to elective colorectal surgery vs controls (*n* = 90)	3 cycles of UL ischaemia with cuff inflation to 200 mmHg (5/5 min)	RIC significantly reduced plasma concentrations of IL-1β, TNF-α up to 3 days post-surgery (*p* < 0.001)

## Humoral pathways of inflammation and cell survival

### RISK, SAFE and HIF-1α

Many studies have demonstrated, that RIC reduces cardio myocyte cell death in I/R and other pathologies [31, 63, 69, 73, 154]. The reperfusion injury salvage kinase (RISK) and survivor activating factor enhancement (SAFE) pathways, are fundamental in protecting the heart from I/R injury [44, 122]. The RISK pathway acts to prevent opening of the mitochondrial permeability transition pore (MTP), when activated before reperfusion [50]. There is a clear role for RIC and protein kinase C (PKC), which has cardioprotective actions in both ischaemia and reperfusion [63]. PKC regulates the opening of the MTP by mediating K_ATP_ dependent channels and controlling calcium influx [51]. The RISK pathway is also activated by adenosine, bradykinin, and sphingosine which bind to receptors on the cell membrane [122]. This leads to upregulation of endothelial nitric oxide synthase (eNOS) and nitric oxide (NO) which induces vascular vasodilatation in the heart and vasculature. Cross-talk exists between the RISK and SAFE pathways to augment cell survival, and this has been demonstrated across different species in studies of RIC and I/R [131].

The SAFE pathway (first described by Lecour in 2009) is an alternative pro-survival axis to RISK. SAFE was found to act on the MTP when ERK/MAPK (RISK) were inactivated and thus its independent actions were demonstrated [44, 88]. SAFE describes the pathway initiated by the binding of TNF-* α* to the plasma membrane and the subsequent activation of JAK/STAT transcription factors [60]. In experimental studies of myocardial I/R, SAFE has upregulated STAT3, (likely via the Sphingosine Kinase 1 enzyme) [44, 79]. In human studies, RIC is associated with the upregulation of myocardial STAT5 [66]. STAT is able to activate NF-κB to influence the MTP and promote cell survival; however, NF-κB is itself pro-inflammatory, and coupled to cytokine secretion and pyroptosis/the NLRP3 inflammasome (Fig. 2). This presents somewhat of a conundrum in the treatment of inflammatory cardiac conditions. JAK/STAT can further evoke ‘notch’ signalling between local monocytes, which is directly linked to increased IL-6 manufacture and downregulation of the anti-inflammatory M2 macrophage/IL-10 [67, 121]. As the SAFE pathway can activate JAK/STAT and NF-κB, it can also promote adverse cardiac remodelling and heart failure [44]. With this in mind, other humoral factors/pathways must be triggered by RIC to account for a reduction in pro-inflammatory cytokines.

The molecule hypoxia inducible factor (HIF-1α) has been linked to pro-survival signalling in I/R [63]. During periods of reduced oxygen tension, HIF-1α mediates a shift in mitochondrial metabolism towards anaerobic glycolysis, which induces production of pyruvate dehydrogenase kinase 1 (PDK1) and limits entry of acetyl-CoA into the TCA cycle. This acts to preserve cellular energy and limit apoptosis [19, 90]. In differentiated macrophages, however, this metabolic change results in increased synthesis of cytokines such as IL-1β *a*nd IL-18 via the NF-κB pathway [19, 143]. This is somewhat paradoxical, as increased levels of HIF-1*α* have been associated with cardioprotection following RIC [59, 147]. It is possible that repeated stimulation of HIF-1*α* causes uncoupling of cytokine synthesis and immune tolerance; as is the case in other TLR4-dependent pathways [6]. It is already established that persistently elevated levels of HIF-1*α* can induce hypoxia tolerance [89]. Again, HIF-1α alone cannot explain the interaction between RIC and cytokine levels observed in animal studies [114, 152].

Stromal-derived factor (SDF-1α/CXCR4) has also been associated with HIF-1α and cardioprotection secondary to reduced apoptosis and upregulation of PI3K/ERK1/2 (RISK). Exogenous SDF-1α protects human myocardium from I/R injury [98] and is released from endothelial cells during RIC [12, 27]. In a rodent model of spinal cord injury, infused SDF-*1α* reduced levels of IL-1β, IL-18, TNF-*α*, and NLRP3 inflammasome production, suggesting that it has anti-inflammatory actions [150].

### NF-κβ and associated inflammatory pathways

NF-κβ is an important molecule and transcription factor, involved in all aspects of inflammation and tumour activity. In addition to stimulation of the NLRP3 inflammasome and pyroptosis (discussed extensively above) it has many anti-apoptotic actions, making upstream inhibition problematic in inflammatory diseases [134, 146]. In response to this observation, strategies such as IL-1 inhibition and caspase-1 inhibition have been proposed in the treatment of cardiac inflammation [1]. NF-κβ is coupled to other proteins such as Iκβ (inhibitor of kappa beta) which enable self-regulation. Well-known anti-inflammatory drugs such as glucocorticoids e.g. dexamethasone can interact with inhibitors of NF-κβ, to reduce cytokines and the inflammatory response [146]. It is interesting then to note that RIC has also modified Iκβα proteins in a rodent model of acute lung injury, leading to reduced activity of NF-κβ and reduced TNF-α, IL-1β and IL-6 secretion [76].

Several other studies have proposed that RIC can suppress the TLR4/NF-κB/inflammasome axis and reduce cytokine secretion [74, 130]. Moreover, there are multiple pathways that are linked to NF-*kB* which are mediated by TLR receptors, and HMGB1 [3, 121]. Activity of the JAK/STAT pathway has been coupled to RIC-mediated cytokine modification as described [15, 124]. The receptor of advanced glycosylation end-products receptor (RAGE) pathway is associated with inflammasome production and HMGB1, (Fig. 2). Activation of RAGE is pro-inflammatory, and this can also downregulate RISK [124]. In mice undergoing RIC following myocardial ischaemia (RICPost), a decrease in infarct size was associated with a decrease in cardiac RAGE expression and levels of HMGB1 [142]. This may indicate that (as yet unidentified) humoral factors stimulated by RIC, can inhibit RAGE. The evidence at present for this is limited and further research is required.

To summarise, further humoral intermediaries may exist to link the vascular phenomenon of RIC to the above inflammatory pathways, and the findings of pre-clinical studies cannot be explained by RISK/SAFE alone. It seems most feasible, that such circulating anti-inflammatory factors might originate from the local trigger vessel; however, it is also recognised that there is a role of regional and distant vasculature [123]. Identifying these intermediate compounds and their mechanisms, remains a priority; as targeting both pro-survival and anti-inflammatory pathways in synergy could result in maximum cardiac protection.

### Micro and macrovascular humoral factors

Following myocardial I/R, an increased number of neutrophils in the resistance vessels contribute to local vasoconstriction, microvascular obstruction and ‘no reflow’ [57]. Meanwhile, there is further immune cell influx (including mast cells), platelet activation and upregulation of the clotting cascade via tissue factor and Von Willebrand’s factor [56, 101]. Cytokines induce disruption of endothelial tight junctions and this culminates in leakage from capillaries into the extracellular space and the concurrent presence of micro-vessel haemorrhage and thrombi. Both no reflow and MVO post STEMI are considered prognostic, and this relationship is independent to infarct size [82]. It is, therefore, important to investigate ways to target this phenomenon [57]. In a large clinical trial of 696 STEMI patients (LIPSIA CONDITIONING), neither RIC alone nor in combination with post-conditioning following PPCI, demonstrated any reduction in MVO following cardiac MRI. There was, however, a significant improvement in myocardial salvage index in the cohort who received RIC and post-conditioning in combination (*p* = *0.02*) [32].

Despite these results in larger clinical trials, RIC has increased both macro and microvascular flow on ultrasound Doppler, in two studies [85, 95] and improved forearm blood flow (venous occlusion plethysmography) in healthy male volunteers [47]. Both RIC and NO donors such as glycerin trinitrate (GTN) improve endothelial-mediated dilatation and reduce vascular reperfusion injury. Given that GTN in combination with RIC confers no additional endothelial protective benefit, it is hypothesised that conditioning utilises NO pathways to induce vasodilation and organ protection [47]. The vascular release of extracellular vesicles and exosomes in response to RIC is further considered below.

## Does RIC mediate innate immune cell activity?

### NETs and neutrophil recruitment

Neutrophils are a considerable driver of the inflammatory process, and are responsible for significant cytokine release and tissue damage [34, 111]. Activated neutrophils release neutrophil extracellular traps (NETs), which are structures containing DNA and histones, amongst other inflammatory molecules. Neutrophils are stimulated to release NETs following exposure to alarmins and defensins, and this is mediated by neutrophil elastase (NE), myeloperoxidase (MPO) and activated platelets, which bind to cellular HMGB1 receptors. NETs can induce host cell death in response to immune invasion, in a slow apoptotic process called NETosis [10, 104, 111]. NETs can also cause inflammatory obstruction within the microvasculature, contributing to MVO. NETosis is associated with worsening outcomes post STEMI [54].

The impact of RIC on neutrophils, adhesion molecules and enzymes such as MPO, has predominantly been investigated in animal models of I/R injury, endotoxemia and acute kidney injury [30, 69, 76]. In rats undergoing RIC by femoral artery occlusion, prior to 45 min of left renal ischaemia, RIC significantly reduced expression of ICAM-1 adhesion molecules, in addition to pro-inflammatory cytokines (TNF-*α*, IL-1β) [69]*.* In humans with ulcerative colitis and moderate disease activity, however, RIC did not reduce neutrophil infiltration or other markers of inflammation such as CRP, following rectal biopsies [39]. Conversely, in a large randomised control trial (*n* = 206 participants) of patients undergoing ablation for atrial fibrillation, RIC significantly reduced neutrophil–lymphocyte ratio and levels of CRP up to 48 h post-operatively (*p* < 0.05) [71]. A recent review has recognised neutrophils as important targets in cardioprotection [5], especially considering that these cells have the potential to polarise macrophages and enhance the acute inflammatory response.

### Lymphocytes, monocytes and splenic response to RIC

In animals, RIC influences both circulating leucocytes and immune precursors in the spleen in models of cerebral ischaemia [14, 96]. In rats undergoing middle cerebral artery infarction, RIC was associated with increased splenic volume and lymphocytes, with reduced cytotoxic T cells and natural killer cells (NK) in cerebral tissue at day 3 [14]. These changes were negated when animals underwent splenectomy, suggesting an underlining mechanism of splenic conditioning. Similarly, in a second study, RIC increased a colony of non-inflammatory monocytes (CD43^+^/CD172a^+^), in addition to increasing circulating B lymphocytes [96]. This is interesting given that in infection and immunity, B lymphocytes are central to immune conditioning and ‘immunological-memory’ [77]. Others have proposed that RIC utilises a ‘splenic-vagal nerve’ axis of cardioprotection, given that the cardioprotective effects of RIC are abated in animals undergoing splenectomy and vagotomy. Splenectomy is associated with reduced amounts of STAT3 (SAFE pathway) but not all related humoral factors have been fully identified [62, 93]. It is proposed that the anti-inflammatory cytokine, IL-10 may be important in an RIC-mediated splenic axis of cardioprotection [62].

### Platelets and the coagulation cascade

With respect to platelet function and the coagulation cascade, animal models have demonstrated the fibrinolytic and anticoagulant benefits of RIC [97]. However, this benefit has not been observed in humans [41, 46, 52, 116]. Following the ERIC-PPCI/CONDI-2 trial, a subsequent sub-analysis of the study population was performed to look for fibrinolysis benefit, but no firm trends were observed, with the exception of a reduction in time to thrombosis at 48 h [41]. However, a recent study of patients with underlining coronary artery disease has demonstrated that whilst RIC cannot influence platelet aggregation alone, when combined with dual anti-platelet therapy (DAPT) in vitro, there is significant de-activation of collagen-dependent, platelet glycoprotein integrin molecules [87]. Further studies showing clear fibrinolytic benefit post-RIC vs controls are, however, necessary to reaffirm this.

## Extracellular vesicles (EVs) in inflammation and immunity

It has long been proposed that the organ-protective effects of RIC can be attributed to the release of humoral factors by the ‘trigger vessel’, which reach the target tissue to reduce inflammation and cell death. Recently, endogenous nanoparticles known as exosomes have been thought to facilitate this transfer, perhaps aided by an improvement in vascular flow, secondary to the release of vasoactive compounds [28, 35, 38, 139].

Exosomes represent the smallest size of extracellular vesicles (measuring 50–100 nm in diameter) and have a wide variety of functions in ischaemia and inflammation. Such nanoparticles can be derived from many types of cells including endothelium, haematopoietic cells and platelets, and their function is defined by the underlying pathology and cell of origin [22]. Exosomes carry chemokines and genetic material such as microRNA, which permits distant genetic transcription and cellular cross-talk. Such exosomes engage with target cells using a range of surface molecules expressed on their lipid bi-layer including tetraspanins, annexins, integrins and receptors of the major histocompatibility complex (MHC) [18, 26, 55]. They are distinct from other small extracellular vesicles and apoptotic bodies as they are smaller and carry different contents; which can be both anti and pro-inflammatory, depending on their stimulus [22].

It is necessary to define which contents may be most implicated in the inflammation of I/R and other conditions (Table.3). Exosomes carrying microRNA-21 (miR-21) have been identified in two recent RIC studies [35, 110] as limiting apoptosis and infarct size, respectively. In a rodent model of endotoxemia induced by LPS, mice undergoing RIC prior to caecal puncture were found to secrete organ-protective exosomes carrying miR-21, which mediated HIF-1α and led to cytokine attenuation (reduced levels of IL-6 and TNF-α) [110]. In addition to apoptosis and cytokine release, it has been demonstrated that endothelial-derived exosomes can mediate angiogenesis (via VEGF and eNOS) in response to RIC [16], and, therefore, promote cytokines and endothelial growth factors. Again, this illustrates their breadth of function, in different pathological conditions.

**Table 3:** Exosomes released in response to RIC in recent human/animal studies

Authors	Treatment group	Exosome content/pathways	Inflammatory/therapeutic action
Haller et al. (RCT) [45]	*N* = 32 patients with STEMI undergoing forearm RIC prior to PCI vs controls PCI alone	Platelet-derived EV’s (Calcein/AM^+^/CD41^+^) Endothelial-derived EV’s Leucocyte derived EV’s (CD14, CD66b)	Pro-thrombotic—no differences observed between RIC vs controls ? Pro/anti- inflammatory – monocyte derived micro-particles elevated in RIC group at 1-month post STEMI, otherwise no differences observed
Cui et al. [20]	SH-SY5Y neuronal cells incubated with venous serum of healthy male volunteers following forearm RIC	mi-RNA-126	Increased tolerance to oxygen/glucose deprivation via downregulation of DNMT3B
Chen et al. [16]	Rats undergoing bilateral hind limb RIC prior to LAD occlusion donated plasma to recipient animals in a model of myocardial infarction, and to CMVECs in an in vitro H/R model	HSP70/HIF-1α/eNOS/iNOS/ANG-1/VEGF	Improvement in LVEF in vivo Increase in angiogenesis and reduction in apoptosis in vitro
Li et al. [91]	Mice undergoing bilateral hind limb RIC donated plasma exosomes to recipient mice pre- dMCAO	HIF-1α	Reduction in cerebral infarct size
Pan et al. [110]	Mice undergoing RIC by bilateral femoral artery clamping 24 h prior to caecal puncture, donated plasma to recipient mice with LPS challenge	mi-R21/HIF-1α	Improvement in survival Reduced plasma creatinine levels Reduced serum IL-6, TNF-α which was abrogated in mi-R21 knock-out mice
Minghua et al. [103]	Rats undergoing bilateral hind limb RIC prior to LAD occlusion, donated plasma to H9c2 cells in vitro and to recipient animals in vivo	mi-R24/Bim	Reduced apoptosis and caspase-3 in vitro Reduced infarct size in vivo
Frey et al. (RCT) [35]	*n* = 58 patients with IHD underwent left arm RIC prior to and during isoflurane/sufentanil anaesthesia for CABG, exosomes were sampled and analysed at up to 1 hr post induction	mi-R21, mi-R28, mi-R320, mi-R92a	Reduction in post-operative troponin; however, not known whether related directly to mi-R21
Wider et al. [144]	Both normoglycemic and diabetic rats underwent bilateral limb RIC prior to LAD occlusion and reperfusion. Plasma was donated and incubated with HL-1 cardiomyocytes exposed to H/R	? Apo lipoprotein B-100; C4 complement (multiple proteins identified)	Reduced apoptosis in vitro The above attenuated in diabetic rats

*STEMI* ST-elevation myocardial infarction, *PCI* percutaneous coronary intervention, *EV* extracellular vesicles, *LPS* lipopolysaccharide, *dMCAO* distal middle cerebral artery occlusion, *I/R*ischaemia/reperfusion, *LAD* left anterior descending, *H/R* hypoxia/reoxygenation, *HIF-1α* hypoxia inducible factor 1 alpha, *HSP70* heat shock protein 70, *eNOS* endothelial nitric oxide synthase, *VEGF* vascular endothelial growth factor, *ANG-1* angiopoietin 1, *LVEF* left ventricular ejection fraction, *CABG* coronary artery bypass graft, *IHD* ischaemic heart disease

Regarding the anti-inflammatory actions of exosomes, (reductions in apoptosis and cytokine release), many of these benefits were negated in a study of diabetic rats vs normoglycaemic animals [144]**.** This suggests that animals with underlining co-morbidities and endothelial dysfunction, are unable to generate effective vesicles. However, when receiving exosomes from non-diabetic rats they can be rescued and protection is conferred [25, 144]. The authors of the latter study, make the important observation, that although RIC appears to generate cardioprotective exosomes in vivo, similar benefits can also be derived from the exosomes of control animals in absence of pre-conditioning [144]. This is re-enforced by a recent clinical study of patients undergoing RIC prior to treatment for STEMI, where no significant differences were found in the release of platelet-derived extracellular vesicles or other leucocyte derived vesicles in the intervention group [45].

The study did, however, suggest that whilst there was no increase in cardioprotective vesicles, there was also no increase in pro-inflammatory EV’s. The trial was limited by an absence of inflammatory and traditional end points in cardioprotection, such as infarct size and CRP. It is also noted that larger extracellular vesicles, as opposed to exosomes, may also be more likely to carry pro-inflammatory chemokines [22].

## Does RIC reduce inflammation? Summary

There was never a more appropriate time in this COVID-19 era, to consider therapies which can treat the over-activity of the innate immune response and hyper-inflammation [112, 127]. Considering the above evidence, it is clear that RIC has anti-inflammatory benefits in vivo, across a wide range of different pathologies, at least in animal models. Perhaps the strongest evidence relates to the effects of RIC on pro-inflammatory cytokine release in endotoxaemia [68, 74, 110]; however, this has also been observed in models of myocardial and cerebral infarction (Table.1). In animals, it is proposed that RIC mediates inflammation by cytokine inhibition, regulation of anti-apoptotic pathways and possibly the reduction of NLRP3 inflammasome production and pyroptosis.

Most evidence from animal studies demonstrates that RIC is able to inhibit NF-κB related cytokine release, either by TLR4 receptor pathways, or other currently undefined mechanisms [134]. Further work is required to establish whether there is a clear link between RIC and other known mechanisms of cytokine release in inflammation, such as the RAGE pathway. It is recognised that, as a result of the nature of the NF-κB pathway, cell survival and cytokine release are closely related [99, 134] and, therefore, it can be difficult to establish if reduced cytokine concentrations are secondary to reduced cell death. Nevertheless, cytokine inhibition in cardioprotection remains a desirable goal, with prognostic value [4, 120].

Previous literature has discussed that an increase in the levels of HIF-1α in response to ischaemia, can stimulate affected tissue to maintain metabolic function upon further hypoxic insult [19]. As RIC has been shown to upregulate HIF-1α, it might, therefore, be suggested that this could induce hypoxic tolerance of both vascular endothelium and target tissue. Both HIF-1α and SDF-1 limit apoptosis following RIC [27, 63]. Shear stress and mechanical stimulation of the trigger vessel, induce flow-mediated dilatation (via NO/adenosine/COX), and stimulate the release of exosomes carrying chemo-active compounds to target tissue [38, 85, 95]. Exosomes can also carry pro-inflammatory compounds and chemokines in addition to cardioprotective substances, and are, therefore, ‘a double-edged sword’ in inflammation [45, 55]. Despite this, the aforementioned studies investigating RIC and exosome release have reported protective effects (Table.3).

Although there is some evidence that RIC can modulate immune cell response in animal studies (e.g. neutrophil/lymphocyte ratio) the authors concede that there is a lack of consistent clinical data. The role of immune cells in cardioprotection is an emerging and novel field on which to base further work, and the effects of RIC should continue to be investigated. Other potential immune targets such as fibroblasts, pericytes and mast cells have also been identified for further study [5]. Consistent with other aspects of RIC, it would be misleading to suggest that the anti-inflammatory effects have proven profound in humans, although there is a lack of large-scale focused RCT’s in patients with hyper-inflammation. Moreover, a select few studies have offered some hope that under the right circumstances, clinical translation could be achieved [53, 140]. A further step in addressing this might be to consider whether we have measured inflammatory outcomes in the correct clinical setting to date.

## Future considerations: towards a higher risk patient cohort

Reflecting on clinical challenges to date, it is suggested that the impact of both baseline and peak inflammation in clinical trials of RIC has been underestimated. For example, individuals with chronic inflammatory disease and persistent low levels of inflammation at baseline, may already be resistant to remote conditioning [39]. The exact reasons for this remain elusive, but may be related to persistent endothelial activation, chronic cytokine release and defective exosome/humoral factor production, (as observed in diabetic animals) [25, 144]. With respect to the STEMI patients of CONDI-2/ERIC-PPCI, it is possible that the outcomes do not represent a failure of RIC to show significant benefit, but instead a success of modern primary percutaneous cardiovascular intervention (PPCI) in this cohort [11, 49]. I.e. it is not clear whether the ‘inflammatory peak’ following successful PCI, was significant enough to demonstrate an improvement in the primary outcome measures of the trial (cardiac death/ hospitalisation at 12 months) [48].

As proposed by several authors [11, 49, 65], higher risk patients with amplified inflammatory response to STEMI, might be proposed as the appropriate target for RIC e.g. those with large anterior infarcts who are *late presenting*, patients in cardiogenic shock, out of hospital cardiac arrest, those who develop angiographic no reflow of a large culprit vessel and those who are only able to receive thrombolysis and not primary PCI [49, 81]. Global inflammation is observed in patients with endotoxaemia and viral infection—these patients may also be favourable candidates for RIC [43]. Moreover, remote conditioning in combination with pharmacotherapy may be of benefit in preventing the development of a cytokine storm.

Given the challenges in clinical translation [65], RIC should be trialled as an adjunctive therapy in combination with gold-standard treatments in the above ‘*high-risk’* cohort. It has been demonstrated above, that RIC can act synergistically to reduce inflammation when combined with pharmacotherapy and activation of neuronal pathways [24, 33, 141].

The difficulty in predicting the timing of a major inflammatory insult remains a significant dilemma for human interventional studies. However, given the reproducibility and extent of the survival benefits observed in animal models; the anti-inflammatory effects of RIC warrant further clinical pursuit.
